# A User-Centered Approach: Understanding Client and Caregiver Needs and Preferences in the Development of mHealth Apps for Self-Management

**DOI:** 10.2196/mhealth.7136

**Published:** 2017-09-26

**Authors:** Roxanna M Bendixen, Andrea D Fairman, Meredith Karavolis, Carly Sullivan, Bambang Parmanto

**Affiliations:** ^1^ School of Health and Rehabilitation Sciences Department of Occupational Therapy University of Pittsburgh Pittsburgh, PA United States; ^2^ School of Health and Rehabilitation Sciences Department of Occupational Therapy MGH Institute of Health Professions Boston, MA United States; ^3^ Department of Occupational Therapy Duquesne University Pittsburgh, PA United States; ^4^ School of Health and Rehabilitation Sciences Health and Information Management University of Pittsburgh Pittsburgh, PA United States

**Keywords:** mobile health, telemedicine, self-care, adolescence, spina bifida, cerebral palsy, spinal cord injury

## Abstract

**Background:**

Many adolescents and young adults with chronic illness or disability often fail to develop the self-management skills necessary to independently handle medical and self-management routines. In light of these needs, we are developing iMHere 2.0 (Interactive Mobile Health and Rehabilitation), a mobile health (mHealth) system to support a self-management program.

**Objective:**

Our objective was to gather data from persons with brain and spinal cord anomalies (BSA) and their caregivers to better understand how mHealth would be most helpful in supporting them to proactively manage daily self-care routines and to access medical care as needed. The specific purpose was not only to gather feedback and to gain increased insight into the design of the new version of iMHere, but also to gather perspectives of new groups, namely adolescents as young as 12 years and their parents and/or caregivers.

**Methods:**

Our project employed focus group sessions and surveys to collect data from participants with BSA, as well as their caregivers. A total of six focus group sessions were conducted on four separate occasions until the data gathered reached saturation. The objectives of our focus group sessions were to better understand ways to develop mHealth systems to support self-management, to promote independence, to motivate long-term system use, and to prevent medical problems that lead to hospitalizations and emergency room visits for youth and young adults with BSA.

**Results:**

A total of 16 youth and young adults with BSA and 11 caregivers participated in the sessions. Within and among our groups, the following five overarching themes emerged from the data: (1) make it easy, (2) engage, (3) educate and prepare, (4) motivate and support, and (5) personalize. Participants shared their perspectives and detailed information about mHealth apps that would be important for independence in self-care and self-management.

**Conclusions:**

Our findings suggest that most individuals keep their mobile phones with them at all times and typically use a mobile phone for social media, music, photos, and texting. Our qualitative analysis indicates that youth and young adults with BSA, as well as their caregivers, acknowledge the importance of being actively engaged in developing and using mHealth apps that monitor and manage their health care needs. Information gleaned from these focus group sessions and surveys have provided data to refine the iMHere 2.0 mHealth prototype platform that we have developed.

## Introduction

### Background

Self-management is a gradual process beginning in childhood, with increasing responsibility during adolescence until one reaches his or her maximum degree of independence in adulthood [1]. Self-management skills and behaviors change over time depending on life circumstances and phases of disease and, therefore, require support strategies tailored to meet these changing needs [2]. Self-management support is the assistance given to people with chronic conditions, which enables them to manage their health on a day-to-day basis. Self-management support can aid and inspire people to learn more about their conditions and to take an active role in their health care [3]. Typically, self-management skills progress during the adolescent and young adult years, which is a developmentally appropriate time in one’s life to seek separation from parents and gain full independence with regard to self-management [4,5]. Unfortunately, many adolescents and young adults with chronic illness or disability often fail to develop the self-management skills necessary to independently handle medical and self-management routines [6-8]. For example, individuals with developmental brain and spinal cord anomalies (BSA), including persons with spina bifida (SB), congenital hydrocephalus, and cerebral palsy, often incur preventable secondary conditions such as pressure ulcers, urinary tract infections, and sepsis [9-11]. These complicated conditions occur most frequently in the youngest age groups and account for more than 30% of hospitalizations [10]. However, proactive management of primary and secondary conditions can have a positive effect on medical outcomes and rates of hospitalization [12]. Currently, mobile health (mHealth) interventions are becoming increasingly important for reducing heath care costs, encouraging proactive self-management skills, and improving well-being [13-18].

In light of the growing need of persons with BSA, we developed iMHere (Interactive Mobile Health and Rehabilitation), an mHealth system to support a self-management program [19]. The initial version of iMHere 1.0 connected adults with disabilities to clinicians and was implemented to support individuals with SB [20] and spinal cord injury (SCI). iMHere 1.0 was developed on an Android platform and did not support iPhone or other non-Android users. Previous surveys, usability testing, and qualitative feedback were gathered from persons with SB and SCI to improve upon the functionality and user interface of iMHere 1.0 [21]. In the process of gathering these data, it was determined that a new version of the system was needed to allow for a cross-platform design to ensure that the system could be used on the iPhone operating system, Android, and other platforms. This design also allowed us to create a new system that is more appealing for sustained use by a younger population and to integrate caregivers and community support. The newer version of iMHere 2.0, currently in development, will have a cross-platform design and will incorporate support for adults and youth with disabilities and chronic conditions, clinical personnel, caregivers, and community support.

### Objectives

The specific purpose of our study was to employ a user-centered approach to gather increased feedback and to gain insight into the design of the new version of iMHere. We also sought to gather perspectives of new populations, namely adolescents as young as 12 years and their paid or unpaid caregivers. Our project employed surveys and focus group sessions with participants with BSA, as well as their caregivers. We chose the focus group method of data collection, which is a technique involving the use of in-depth group interviews to facilitate the generation of deep, rich, and diverse data through the social interaction of the group participants [22,23]. Our focus group objectives were to better understand ways to develop mHealth systems to support self-management, to promote independence, to motivate long-term system use, and to prevent medical problems that lead to hospitalizations and emergency room visits for youth and young adults with BSA. This iterative user-centered design occurred within the development phase of iMHere 2.0 to ensure that the final mHealth system would fulfill the users’ desired functionality and meet their needs.

## Methods

### Participants

Participant (youth and young adults with BSA) and caregiver recruitment for our qualitative focus group sessions occurred through collaboration with human services agencies, including the Spina Bifida Association of Western Pennsylvania (SBAWP), the Community Living and Support Services (CLASS), research registries, and personal referrals. The study received approval from the institutional review board (IRB) of the University of Pittsburgh (Protocol # PRO14120443). iMHere 2.0 is specifically being developed for youth and young adults with BSA, as well as their caregivers. Our recruitment efforts were intended to target youth and caregivers who met the inclusion criteria, but adults with BSA also participated in the study. We sought to achieve a wide range of perspectives and ideas from our focus group discussions through recruiting and enrolling participants with variations in age, gender, living environment, and disease severity. The IRB-approved flyers were emailed to potential participants from our collaborating agencies and posted in the community. Individuals were requested to contact the study coordinator if they met the criteria and were interested in participating in a focus group session. Before participating in the study, written informed consent was obtained from all adult participants and at least one parent or guardian for minor participants. Written assent was obtained from minor participants whenever possible; dissent from all minors was respected (Multimedia Appendices 1 and 2). Inclusion criteria required a diagnosis of BSA or the caregiver of an individual with BSA, the ability to understand and speak English, and willingness to participate in a one-time only focus group interview lasting up to 120 min. Participants received US $25 remuneration for participation, reimbursement for parking (if applicable), and a free meal. Focus group sessions were facilitated by the first and second authors (experienced focus group moderators) between May and October 2015 in central locations most convenient to our participants. Focus group sessions comprised participants and research team members only. Apart from the moderators, a notetaker was present to observe and document nonverbal interactions, verbal exchanges, and general content of the group discussions.

### Focus Group Sessions

A PowerPoint presentation developed by the moderators was used to organize and guide the focus groups. The PowerPoint slides also served as a means to provide screenshots of the existing version of the iMHere app in development for participants to view. Guided focus group sessions lasted approximately 120 min and were audiorecorded and professionally transcribed. Each focus group session began with a broad definition of mHealth technology and self-management and a request to participants to individually discuss their experiences with using mobile devices and apps (Multimedia Appendices 3 and 4). Our research objective was to better understand ways to develop mHealth systems to support self-management for youth and their caregivers; therefore, guided focus group questions centered on areas such as (1) examples of how participants have used mHealth apps in the past; (2) what apps have they found beneficial or unfavorable and why?; (3) what resources participants may need to better support their health?; (4) feedback on mHealth designs to help motivate and engage users; and (5) overall expectations for technology to support their wellness and prevent medical complications. Focus group sessions ended with the collection of demographic data, along with a follow-up survey requesting additional comments regarding the mHealth functions, importance of the functions for promoting self-management and independence, as well as personal experience with technology. Following each focus group session, the research team met for a debriefing session to share information and to discuss observations and potential ways to improve the focus group process. Field notes and debriefing sessions supplemented the participants’ oral discussions and enabled a richer analysis of the data [22,23].

### Data Analyses

Final datasets included participant demographics, follow-up surveys, transcribed focus group sessions, as well as fieldwork observations and debriefing sessions. Throughout the research process, the primary author used constant comparison techniques, whereby each focus group session, along with the corresponding fieldwork observations and debriefing sessions, was compared with the previous data and not considered independently. This enabled data to be treated as a whole in lieu of fragmenting the data. Constant comparison also enabled the initial identification of emerging patterns and themes [24].

The use of framework analysis, as described by Ritchie and Spencer [25], provided clear steps to assist in the management and analyses of the data. Initial key patterns within the datasets were first identified by the primary author through a rigorous approach of familiarization through reading and re-reading each transcript on numerous occasions, along with information from the questionnaires, fieldwork observations, and debriefing sessions [26-28]. This was achieved through hand coding datasets line-by-line by marking the data with varied colored highlighter pens to identify important quotes, initial ideas, and concepts arising from the data. Two research assistants, who were not present during the focus group sessions, thoroughly reviewed the datasets independent of the first author and each other, with direction from the second author. Research assistants used cutting and sorting as a formal way of identifying and organizing important quotes. Following the independent identification of ideas and concepts within the datasets, researchers and research assistants came together on three occasions to discuss their findings as a group and to categorize initial codes. Researchers conducted focused coding to eliminate, combine, and divide categories, thereby allowing for the development of patterns within the data. Patterns were then synthesized and reduced to themes [25,26,28,29]. Data relevant to each theme were fully reviewed and discussed by the research team. Themes were refined and rephrased to more clearly describe the *story* being told by our participants and their caregivers. Agreement among the independent reviewers provided confidence that we had identified appropriate themes. Our central research objective shaped our framework and final interpretation [26,29]. Six in-depth focus group sessions were sufficient to achieve data saturation and to enable the development of meaningful themes and useful interpretations [30-32].

## Results

A total of six focus group sessions were conducted on four separate occasions. Initially, 19 youth and young adults with BSA demonstrated interest in participating in focus group sessions; 3 did not show up for the session and hence could not provide assent, leaving a total of 16 youth and young adults with BSA in our focus groups. A total of 15 caregivers (paid and unpaid) contacted the study coordinator and discussed participation. Of these 15, 2 caregivers canceled because of an illness, and 2 caregivers did not show up, leaving a total of 11 caregivers in our focus group sessions (Table 1).

**Table 1:** Demographic characteristics of focus group participants.

Characteristic		Participants (n=16)	Caregivers (n=11)
**Gender**			
	Female	12	5
	Male	4	6
**Age range, in years**			
	12-17	3	0
	18-24	3	0
	25-34	3	0
	35-44	0	0
	45-54	5	1
	55-65	2	10
	Range	15-62	45-64
	Mean (SD)	34.7 (17.4)	51.1(6.6)
**Race**			
	White	15	11
	Asian	1	0
**Ethnicity**			
	Hispanic	1	0
	Non-Hispanic	15	11
**Education**			
	Some high school	4	0
	High school graduate	3	0
	Some college	1	1
	Trade/Technical/Vocational	0	2
	Associate’s degree	0	1
	Bachelor’s degree	7	3
	Master’s degree	1	3
	Doctoral degree	0	1
**Marital status**			
	Single	13	2
	In a committed relationship	3	7
	Divorced/Separated	0	2
**Residence**			
	Urban	7	3
	Suburban/Rural	9	8

Within and among our groups, five overarching themes emerged from the data, which are as follows: (1) make it easy, (2) engage, (3) educate and prepare, (4) motivate and support, and (5) personalize. The *make it easy* theme describes why mobile phone users typically refrain from or give up using apps. The *engage* theme illustrates the importance of having an app that is colorful, consolidated, convenient, and helpful. The *educate and prepare* theme presents our participants’ and caregivers’ views on the importance of having individualized information to inform and educate. The *support* theme is focused on the participants’ consistent descriptions of their desired social needs and personal feedback. Peer and social support was an important and recurring theme throughout our focus group sessions. Finally, the *personalize* theme provides us with thoughtful and innovative ideas to inspire users to adopt the mHealth app for independence and self-management of their personal health and wellness. Table 2 presents examples of the initial concepts which created our codes, the themes that developed from these codes, and the percentage of total participants (youth and young adults, as well as caregivers) who discussed these ideas and concepts throughout our focus group sessions.

**Table 2:** Examples of codes, developed themes, and prevalence of overall participant/caregiver contribution.

Examples of codes	Themes	Participants (n=16), n (%)	Caregivers (n=11), n (%)
Don’t use; Too difficult; Need to update; Difficult to type; Too many levels to get through; Too time-consuming; Too slow; Difficult to read—text to voice	Make it easy	13 (81)	10 (91)
Fun reminders; Free movies/Downloads/Games; Educational challenges; Rewards; Colorful and fun; Trivia of the day; Jokes; Entertaining	Engage	14 (87)	10 (91)
Look up information; Learn about my disease; Help to manage stress, etc; Guidance—understanding; Learn from others; Searchable information; Accurate and reliable; Updated information; Resources in the community; Teach basic skills	Educate and prepare	16 (100)	11 (100)
Social engagement; Share; Feedback from others; Peer support; Supportive quote; Social media focused on the disease; Thumbs up; Notification when meet goals; Provide assistance	Motivate and support	16 (100)	10 (91)
Tailor to individual needs; Customizable information; Individualized tracking, view progress; Health reminders; Personalize coaching; Healthy tips; Customizable avatar; Rankings—compare to others; Personal point system	Personalize	12 (75)	11 (100)

It was evident throughout our focus group sessions with both youth and young adult participants as well as caregivers that mobile phone use is extremely prevalent and is an integral part of their daily lives. As one of our participants voiced, “I would feel naked if I didn’t have my iPhone with me.”

### Make it Easy

#### Participants (Youth and Young Adults With BSA)

Our participants described the difficulty they face when attempting to download and set up a new app on a phone, demonstrating that accessibility is salient in the minds of our respondents. Participants requested options that were “easy to navigate,” “hands free,” “easy to use and reliable,” “fewer keystrokes,” “fast,” “bigger buttons,” and “colorful so that things stand out.” Certain apps they had attempted to use were too difficult and time-consuming to set up. Participants discussed their frustrations with apps needing to be updated or new versions installed, with one young participant summing up their exasperation by stating: “…So I just said ‘forget it’.”

#### Caregivers

Many of our caregivers were sensitive to the difficulties of typing on small phones, setting up apps, and accessing information. One parent stated:

If you think about it, you could make it so it’s a picture rather than needing to read a word. You could make it so that it talks to you rather than needing to read the word.

Caregivers were just as vocal as our young participants about apps that required too long to program, that continuously updated, or that required the input of information more than one time rather than reusing information. Examples included excessive time and effort to input information before an app could even be used. As one caregiver remarked:

I mean, I know I don’t have that great of patience, but just keep it simple, right?

### Engage

#### Participants (Youth and Young Adults With BSA)

Participants discussed apps that they found engaging and interesting and noted that being “convenient, consolidated, and helpful” was appealing to them but also requested bright, colorful, and vivid images. Participants spoke of the ways that an app might “get my attention, if I was busy.” One young adult who occasionally uses basic alarms and reminders stated:

In truthfulness, I don’t always take its reminder. I go on. It’s just that you’re busy in the day, and things happen. You go on to the next thing. Then it rings for 5 minutes. It’ll stop. Then it’ll be over. Then I forget about it.

Ideas discussed focused on the use of “fun” reminders such as visually appealing animations, a specific person’s voice or person laughing, or the use of one’s favorite music. One of our participants suggested that when an alarm or reminder pops up, add a “joke or trivia of the day, famous quotes or something fun; make someone smile.”

#### Caregivers

For many of our caregivers, the features that were suggested to promote engagement focused on ensuring that the mHealth app would serve a useful, relevant purpose while also being “fun and interactive.” Caregivers echoed participants’ suggestions that if the app “brought value to my daily life, was user-friendly, and entertaining...,” it would be used more and more throughout the day.

### Educate and Prepare

#### Participants (Youth and Young Adults With BSA)

Very few of our participants reported using health-related apps on their phones but admitted the benefits of having access to educational resources that were relevant and conducive to their health needs. Current apps on the market were not always pertinent or informative for our particular participants. One young participant stated:

I have the condition of Spina bifida. To have an app that would [easily] go into different sites that focus exactly on that condition so that I can be updated on the newest things that are coming up in terms of medications, and treatments, and that type of thing. That would be cool.

Another participant stated:

I know there’re certain kinds of shunts and stuff. I’m not really aware of what kind of shunt I have, so maybe something that would tell me about that.

Assistance in tracking personal health issues and providing education on health trends was a recurring theme with our participants. Many of the young adult participants specifically mentioned how worthwhile it would be to have personal health-related information on an app, “all in one place” that they could easily access and present to their doctor. Doctors’ appointments can be rushed and the ability to have complete health information or health-related questions available in an organized fashion may increase efficiency in the limited time available.

#### Caregivers

Our caregivers also felt strongly about the provision of educational resources and wanted assurance that the information was “searchable with keywords” and “customizable to that individual.” Caregivers requested automaticity in the ability to “continually update when new guidelines emerge and get a message about the update.” The ability to receive text messages for updates, reminders, and special notices was considered beneficial by our caregivers, who did not want to consistently log back into an app throughout the day to check on important issues. Of great importance was the caregiver’s ability to oversee what educational resources their children had access to. One mother noted:

The parents need input on the information. I can see them thinking they know it all based off what came across the screen. The information should supplement the family, not replace the family, right?

For caregivers of our younger participants, educational requests that were focused on basic skills were suggested. Interestingly, caregivers were seeking information on safety in daily living skills such as the safest way to get in and out of a shower or information on safety within the community such as how to cross the street or efficiently scan the environment. One caregiver talked about the importance of education by stating:

...physical community resources. Accessibility. Places a family can go in the community like parks and things like that, that would be great.

Although caregiver requests for education regarding the diagnosis and basic living skills were common, a number of caregivers also discussed the importance of information regarding insurance and the purchase of medical supplies. The potential of an app that could provide information on where supplies could be purchased for the best price or lowest insurance co-pay, such as a Web-based versus a medical supply store, appeared to be of great value to caregivers. One caregiver suggested:

An app that would help the consumer like me do a little comparative shopping when we’re buying supplies, that kind of thing.

### Motivate and Support

#### Participants (Youth and Young Adults With BSA)

Most of our participants reported using their phones for social media, especially Facebook or Snapchat, and keeping in touch with social acquaintances and family through texting. Social support and feedback was the most consistent and prevalent theme in our youth and young adult–based focus group sessions. Sharing, connecting, and communicating with friends and family to help achieve personal goals was a topic of discussion throughout. One participant mentioned that it would be beneficial to have support for stressful days:

Yes. Deep breaths or just remind me to bring your head back to what you’re doing now instead of worrying about what’s happening later.

Another young participant who echoed the importance of support noted:

...just talking to someone who’s going through the same thing that I am and seeing how they handle it and what they do to calm themselves down and stay sane.

Interestingly, the ability to motivate others through the meeting of one’s goals was also a frequent topic. One participant said:

I think if you were to give informational tips...that you could share with your friends and family, like healthy tips based on what milestones you’ve reached through using the app—being able to share how you were successful so that they can also be successful.

Participants talked at length about social connection. The ability to share goals and milestones with friends and family and to receive feedback or advice on those goals and milestones was a consistent theme. Participants varied in their suggestions on feedback from something as simple as “a little emoticon that’s active or that shows an expression or energy level as you’re meeting the goals” to more direct feedback such as “Great job using that app for your medications!”

#### Caregivers

Caregivers were invested in having the information they needed so that they were fully able to provide support and feedback to their children/clients. According to a caregiver, such information should include:

...a report regarding missed medications or not responding to flags, poor health reports.

A few caregivers were focused on medication management and wanted the ability to use an app “to scan the barcode on the medication bottle,” or for information such as prescribed dosage, side effects, or potential interactions among medications prescribed. Caregivers were also invested in providing feedback to their children when they were “doing a great job on using the app.” One caregiver requested:

maybe we could have a summary of the data and what they can be praised for, like “Hey great job on keeping your schedule this week”; making it positive for them.

### Personalize

#### Participants (Youth and Young Adults With BSA)

An essential component for any health-related app or device that is focused on personal health and wellness is to “know the user.” The ability to access user-relevant information and set personal goals was a discussion point with our young participants as well as the ability to add or remove information. One participant noted:

...add information or take away information because health and different things come and go with our history. It should be customizable for that individual person.

Our participants were also acutely interested in visually following their progress on individualized goals and being able to share that process with others. Tracking progress from start to finish through the use of reports or spreadsheets appeared to be extremely useful for the participants. The ability to compare personal outcomes over time and compete with themselves was exceptionally appealing to our young participants. One participant stated:

So be able to see your progress in compiled form so you can judge where you are and compare it to where you wanna be.

A novel idea for personalization and to provide feedback on meeting health-related goals was the use of a customizable avatar—an avatar that matures, develops, and changes as the client learns more, achieves more goals, and becomes more independent in their self-management skills. One of the participants said:

You can have a little avatar–that would be cool

Additionally, incorporating monetary and tangible rewards for meeting goals was also relevant to our participants. One participant stated:

Somehow the points that you would earn for doing healthy things then could be converted into a gift certificate or something you really want.

Ideas for material rewards included a downloaded movie or music (with less focus on games), money or gift cards, candy, and restaurant or store gift certificates.

#### Caregivers

Caregivers understood the importance of providing specific feedback on personal goal attainment and the importance of rewarding “small steps.” Most of our caregivers focused on the use of a point system for rewards and recommended a system that would allow participants to achieve certain points and redeem them with toys, food, certificates, or even stickers.

Finally, a few of our caregivers acknowledged they didn’t “always push for independence; I feel like I’ve held her back some.” Referring to a personalized app focused on self-management and self-care, one of the caregivers noted:

...would be a great idea to help lift some of that pressure off us.

Another caregiver stated:

Using something tailored like this can help them start to gain some of that confidence in being able to do things for themselves.

## Discussion

### Principal Findings

Focus groups were held with youth and young adults with BSA and their paid or unpaid caregivers in our attempt to better understand how mHealth would be helpful to support them in proactively managing daily self-management routines and accessing medical care as needed [12,15]. Participants shared their personal views and provided detailed information about mHealth apps that would be important for independence in self-care and self-management. Our objective was to use the data gleaned from these focus group sessions to refine the mHealth prototype platform that we have developed, called iMHere 2.0, an app with a suite of modules that can be personalized and tailored to an individual’s needs.

Our findings suggest that most individuals keep their mobile phones with them at all times and typically use a mobile phone for social media, music, photos, and texting. This is consistent with a previous study on the use of cellphone in the general population [33]. This is also good news because the “always carry, always on” connectivity can be harnessed by mHealth apps such as iMHere to support self-management.

On the basis of participant and caregiver comments, a few mHealth-related apps were typically used. Some participants discussed the occasional use of mHealth apps, such as alarms for medical reminders or the use of diet or fitness apps, but consistent or daily use was rarely occurring. In fact, many of our participants voiced how they often ignored basic alarms and suggested the use of something more appealing such as their favorite music, a loved one’s voice, or even a joke of the day when they responded to their reminders. Participants also reported concerns about apps taking too long to program and being difficult and time-consuming to use, along with their frustration with continuous updates.

Recommendations from our participants focused on supporting personal health and wellness and included reminders for attending doctors’ appointments, taking medications, and ordering medical supplies, which is consistent with past research [13,15,17]. For our young adult group, suggestions for increasing independence included education on their diagnosis and medical management, as well as stress reduction, time management, and social relationships. One unique feature of iMHere 2.0 is the ability to deliver care according to various clinical practice guidelines. Providing care guidelines that are relevant, customizable, continually updated, and easy to access through the use of basic keywords was an essential component for caregivers. Additionally, tracking health trends and receiving information based on those trends was a common topic of discussion. The ability to receive basic guidance or advice for self-management based on the information being collected through the mHealth app seemed to increase a sense of independence for the participants. The security of knowing that a clinician or provider was available if such self-management strategies were not successful was essential. An additional benefit included allowing their doctor to have access to these records and to view trends before a health care appointment.

The ability to develop personal self-management goals and to visually track progress toward those goals was also a function that appeared relevant to our focus group participants. Moreover, personalized coaching was an important component to assist with goal attainment and to provide affirmation and feedback. Interestingly, the greatest motivator for long-term use of the mHealth system was not tangible (ie, money, games, and free movie), instead it was the ability to engage with peers and family and share experiences regarding goal attainment and milestones. Sharing their successes so that others may learn about and acknowledge their accomplishments appeared to be exceptionally motivating. Social support and social recognition was consistently discussed as a reinforcer for the development of self-management skills.

### Limitations

It’s important to note that there are limitations in our focus group methods. As recruitment strategies were mainly carried out through our existing collaborations with human services agencies and personal referrals, participants not involved with these agencies may not have been fully represented in the focus group sessions. Therefore, our participants’ experiences may not be universally representative of all youth/young adults and caregivers in the BSA community. In addition, our inability to recruit minority participants limits our findings. It is also important to mention that our focus group sessions were held in closed meeting rooms, and not in natural environments, which may have changed the behavior of some of our participants. As our focus group sessions were one-time only with each participant, data collection techniques only included fieldwork observations, follow-up surveys, and debriefing sessions with the focus group moderators. Each of the authors was directly involved in all focus group sessions and analyses of the data, which may elicit bias into the study and may have shaped the interpretation and understanding of these data.

### Conclusions

These focus group sessions informed refinements in the iMHere 2.0 system. For example, we are “making it easy” through automatic updates, accessibility preferences, and simplicity in the navigation of the system. We are “engaging” youth, young adults, and caregivers through color coding and special icons within iMHere 2.0 and using a variety of personally selected avatars and sounds for alarms. We are “educating and preparing” through the incorporation of resources that are inclusive of medical and psychosocial elements of the diagnosis and that are searchable and customizable for each individual. We are “motivating and supporting” through the ability to text within the system and to receive feedback and encouragement on goals and self-management tasks. We are “personalizing” through customizing the app for each user’s needs, tracking progress on individual goals, preparing reports on health care trends, and providing a rewards center for earning points when using the app to manage individualized health care needs.

Our qualitative analysis indicates that youth and young adults with BSA, as well as their caregivers, acknowledge the importance of being actively engaged in developing and using mHealth apps that monitor and manage their health care needs. Future research will continue to refine iMHere 2.0 through usability studies, which will lead to a longitudinal clinical trial. Through the use of focus groups, our findings will allow us to move forward with quantitative procedures that are meaningful and appropriate for our population.

## Appendix

Multimedia Appendix 1 Informed consent form - youth.

Multimedia Appendix 2 Informed Consent Form - caregivers.

Multimedia Appendix 3 Focus group guide - youth.

Multimedia Appendix 4 Focus Group Guide - caregivers.

## Abbreviations

ACL: Administration for Community Living
BSA: brain and spinal cord anomalies
CLASS: Community Living and Support Services
HHS: US Department of Health and Human Services
iMHere: interactive Mobile Health and Rehabilitation
IRB: institutional review board
mHealth: mobile health
NIDILRR: National Institute on Disability, Independent Living, and Rehabilitation Research
SB: spina bifida
SBAWP: Spina Bifida Association of Western Pennsylvania
SCI: spinal cord injury
