# Supplementary material for: A User-Centered Approach: Understanding Client and Caregiver Needs and Preferences in the Development of mHealth Apps for Self-Management
Source: JMIR Mhealth Uhealth. 2017 Sep 26;5(9):e141. doi: 10.2196/mhealth.7136 (PMC5635231; doi:10.2196/mhealth.7136)
Supplement: Multimedia Appendix 1 [file mhealth_v5i9e141_app1.pdf]

University of Pittsburgh
School of Health and Rehabilitation Sciences
Department of Health Information management

CONSENT TO ACT AS A PARTICIPANT IN A RESEARCH STUDY: PATIENTS

TITLE: MHealth Self-Management and Support System for Chronic and Complex Health Conditions (Component 1- Refinement and Enhancement - FOCUS GROUP)

<u>FOR PARTICIPANTS YOUNGER THAN 18 YEARS OLD</u>

PRINCIPAL INVESTIGATOR:
Andi Saptono, PhD
Assistant Professor
University of Pittsburgh
Dept. of Health Information Management
6023 Forbes Tower
Pittsburgh, Pennsylvania 15260
Telephone: 412-383-5101

CO-INVESTIGATORS:

Brad E. Dicianno, MD
Associate Professor
University of Pittsburgh Medical Center
Dept. of Physical Medicine & Rehabilitation
Kaufmann Medical Bldg, Suite 202
3471 5th Avenue
Pittsburgh, PA 15213
Telephone: (412) 648-6666

Bambang Parmanto, PhD
Professor
University of Pittsburgh
Dept. of Health Information Management
School of Health & Rehabilitation Science
6026 Forbes Tower
Pittsburgh, Pennsylvania 15260
Telephone: 412-383-6649

Roxanna Bendixen, PhD, OTR/L
Assistant Professor
University of Pittsburgh
Dept. of Occupational Therapy
School of Health & Rehabilitation Science
5025 Forbes Tower
Pittsburgh, Pennsylvania 15260
Telephone: 412-383-6603

SOURCE OF SUPPORT: National Institute on Disability and Rehabilitation Research

Why is this research being done?
Researchers involved with this project are interested in improving technology that they have developed to help individuals with chronic and complex conditions manage their medical problem. By better understanding chronic conditions and disability, and opinions of potential users of the software, researchers can then work to improve the software. The purpose of this study is to find out what features and functions of the technology would best support individuals with chronic condition prepare for adult life and take better care of themselves.

Who is being asked to take part in this research study?
Your child is being invited to take part in this research study because they have a disability or chronic health condition. We would like them to take part in a discussion with other individuals with similar conditions on how to best support and prepare individuals with disabilities and chronic conditions to take better care of themselves using technology.

What procedures will be performed for research purposes?
If you decide to let your child to take part in this research study, they will be asked to take part in a group discussion with other individuals with chronic condition, fill out a questionnaire and/or participate in an interview. They may be asked to review a mobile health support system and provide feedback for improvements.

The session will take about <u>60 minutes</u>. The sessions will be audio recorded and later transcribed. This audio recording will allow us to obtain more detailed information about your child's responses than the handwritten notes that will be taken by investigators, and it will allow us to double check our data for accuracy. Basic information about your child such as age and medical problems will be collected. The system will be revised based on their feedback and in the future they may be asked to participate in another round of testing. A member of the research team may call them to clarify any information that is missing or unclear.

University Of Pittsburgh Institutional Review Board | Approval Date: 2/26/2016 Expiration Date: 2/25/2017 | IRB #: PRO14120443

We want to protect your/your child's privacy, so we will not send their name, address, telephone number or other personal identifiable information to anyone. Their personal data will remain protected within the Department of Physical Medicine and Rehabilitation, and will only be accessed by trained members of the research staff.

Neither you, nor your insurance provider, will be charged for the costs of any of the procedures performed for the purpose of this research study. You will be charged, in the standard manner, for any procedures performed for your routine medical care.

What are the possible risks, side effects, and discomforts of this research study?
The risks of participating in this research are minimal. Your child may experience fatigue when answering questions in the interviews or during the group discussion. They will be allowed to take breaks during the interview.

The discussion will be kept *strictly confidential*. The other participants in the group will be asked to keep private what we talk about in the discussion group, but this cannot be guaranteed. There is also a risk of accidental release of your child's information (a breach of confidentiality); however, we will make every effort to prevent this from happening.

Who will know about my participation in this research study?
Any information we gather about your child from this research study will be kept as confidential (private) as possible. To keep their information private, their name will not be used in any publication of research results unless you sign a separate release form giving your permission. In unusual cases, their research records may need to be released in response to an order from a court of law. If the researchers learn that you/your child or someone with whom you know is involved in serious danger or harm, they will need to inform the appropriate agencies as required by Pennsylvania law. Other people who may know of your child's participation are other participants in the discussion group, and medical staffs that work in the clinic where your child is seen. This would include people such as nurses, nursing assistants and the medical secretaries. It is also possible that authorized representatives of the University of Pittsburgh Research Conduct and Compliance Office may inspect your child's research records as part of the institution's routine oversight of research

The information that we gather about you child on paper, such as this consent form, or audio files recorded on tape will be kept in a locked cabinet in the Department of Physical Medicine and Rehabilitation at the University of Pittsburgh. Their research file will be assigned a specific identification number or case number. The information linking the case number to your child's personal information will be stored in a separate, locked location within the Department of Physical Medicine and Rehabilitation. Any electronic information, (information gathered about your child and typed into a computer or electronic audio files), that does not contain their personal and identifiable information will be kept by the Department of Health Information Management at the University of Pittsburgh. The electronic information will be stored on restricted access, password protected servers. Trained researchers, who are directly working on this project, will have access to your child's personal information. Their records will be kept for 5 years past age of majority (age 23 per PA State law) after study participation ends.

Your research data may be shared with investigators conducting similar research. However, this information will be shared in a de-identified manner.

What are possible benefits from taking part in this study?
There are no direct benefits for your/your child's participation in this research study. You/your child may, however, gain the satisfaction of knowing that the information gained from this research could help to improve the healthcare of individuals with chronic conditions and disabilities. However, such a benefit cannot be guaranteed.

Will I be paid if I take part in this research study?
Your child will receive up to $25 as compensation for their time and effort to participate in this study.
Your child may be asked to participate in future rounds of the study. They will be separately compensated for their participation in each round of the study in the same terms described above.

Is my participation in this research study voluntary?
Your child's participation in this research study is completely voluntary. Whether or not you decide to allow your child to

participate in this research study will have no effect on your/your child's current or future relationship with the University of Pittsburgh, or your/your child's current or future medical care at a UPMC hospital or affiliated health care provider, or your/your child current or future relationship with a health care insurance provider. If there is any question that makes your child feels uncomfortable, they do not have to answer them. They may leave the group discussion at any time for any reason.

May I withdraw, at a future date, my consent for participation in this research study?
Your child's participation in this research study is completely voluntary. You do not have to allow your child to take part in this research study and, should you change your mind, you can withdraw from the study at any time. Your/your child's current and future care at a University of Pittsburgh Medical Center, and your/your child's current and future relationship with the University of Pittsburgh and any other benefits for which you/your child qualify will be the same whether you participate in this study or not.

If I agree to take part in this research study, can I be removed from the study without my consent?
It is possible that your child may be removed from the research study by the researchers if, for example, it is determined that they no longer qualify, or if they do not follow the instructions of the researchers.

Disclosures
One or more of the investigators conducting this research has a financial interest in the software system being evaluated in this research study. This means that it is possible that the results of this study could lead to personal profit for the individual investigator(s) and/or the University of Pittsburgh. Any questions you might have about this will be answered fully by the Principal Investigator, ***Dr. Andi Saptono, at 412-383-5101,*** who has no financial conflict of interest with this research, or by the Human Subject Protection Advocate of the University of Pittsburgh (866-212-2668).

****

PARENTAL PERMISSION FOR CHILD to PARTICIPATE IN A RESEARCH STUDY

Minor Participant's Name (Print): ____

I understand that, as a minor (age less than 18 years), the above named child, is not permitted to participate in this Research without my consent. By signing this form, I consent to allow my child to participate in this research study. During the course of the study, if I have any questions or problems, I may contact the investigators listed in the first page of this document. Any questions about my rights as a research participant will be answered by the Human Subject Protection Advocate of the IRB Office, University of Pittsburgh (1-866-212-2668).

___________________________________ ________________________
Name (Print) of Parent/Guardian Date

___________________________________ ________________________
Signature of Parent/Guardian Relationship to Minor

****

ASSENT (of child, if appropriate):
This research has been explained to me, and I agree to participate.

___________________________________ ______________________
Signature of Child Participant Date

The above person cannot sign for assent because:

____

Verbal assent was given to the person obtaining the consent. _____ Yes _____ No

****

CERTIFICATION of INFORMED CONSENT:
I certify that I have explained the nature and purpose of this research study to the above-named individual(s), and I have discussed the potential benefits and possible risks of study participation. Any questions the individual(s) have about this study have been answered, and we will always be available to address future questions as they arise.

____	______________________________
Name of Person Obtaining Consent (Print)	Role in Research Study
____	__________________
Signature of Person Obtaining Consent	Date

VERIFICATION of EXPLANATION to MINOR:
I certify that I have carefully explained the purpose and nature of this Research to the child-subject in age appropriate language. He/she has had an opportunity to discuss it with me in detail. I have answered all his/her questions and he/she has provided affirmative agreement (i.e. assent) to participate in the Research Registry.

____	______________________________
Name of Person Obtaining Assent (Print)	Role in Research Study
____	__________________
Signature of Person Obtaining Assent	Date

****

CONSENT FOR CONTINUED RESEARCH PARTICIPATION:
(Consent of participant if parental permission was originally obtained due to minor status.)
I understand that I am currently participating in a research study. I further understand that consent for my participation in this research study was originally obtained from my authorized representative because I was under the age of 18. I am now able to provide direct consent for continued participation in this research study.

All of the above has been explained to me and all of my current questions have been answered.
I understand that I am encouraged to ask questions about any aspect of this research study during the continuation of this study, and that future questions will be answered by the investigators listed on the first page of this form. I also understand that any questions I have about my rights as a research participant will be answered by the Human Subject Protection Advocate of the IRB Office, University of Pittsburgh (1-866-212-2668).

By signing below, I agree to continue my participation in this research study. A copy of this consent form will be given to me.

____	_________________________
Printed Name of Participant	Date

____
Signature of Participant

The above person cannot sign the consent because:

____

Verbal consent was given to the person obtaining the consent. _____ Yes _____ No

CERTIFICATION of INFORMED CONSENT:
I certify that I have explained the nature and purpose of this research study to the above-named individual(s), and I have discussed the potential benefits and possible risks of study participation. Any questions the individual(s) have about this study have been answered, and we will always be available to address future questions as they arise.

____	__________________________
Printed Name of Person Obtaining Consent	Role in Research Study
____	__________________________
Signature of Person Obtaining Consent	Date

University Of Pittsburgh Institutional Review Board **Approval Date: 2/26/2016 Expiration Date: 2/25/2017** **IRB #: PRO14120443**
