# Supplementary material for: A User-Centered Approach: Understanding Client and Caregiver Needs and Preferences in the Development of mHealth Apps for Self-Management
Source: JMIR Mhealth Uhealth. 2017 Sep 26;5(9):e141. doi: 10.2196/mhealth.7136 (PMC5635231; doi:10.2196/mhealth.7136)
Supplement: Multimedia Appendix 2 [file mhealth_v5i9e141_app2.pdf]

University of Pittsburgh

School of Health and Rehabilitation Sciences

Department of Health Information management

CONSENT TO ACT AS A PARTICIPANT IN A RESEARCH STUDY

TITLE: MHealth Self-Management and Support System for Chronic and Complex Health Conditions (Component 1- Refinement and Enhancement- FOCUS GROUP)

FOR CAREGIVERS

PRINCIPAL INVESTIGATOR:

Andi Saptono, PhD
Assistant Professor
University of Pittsburgh
Dept. of Health Information Management
6023 Forbes Tower
Pittsburgh, Pennsylvania 15260
Telephone: 412-383-5101

CO-INVESTIGATORS:

Brad E. Dicianno, MD
Associate Professor
University of Pittsburgh Medical Center
Dept. of Physical Medicine & Rehabilitation
Kaufmann Medical Bldg, Suite 202
3471 5th Avenue
Pittsburgh, PA 15213
Telephone: (412) 648-6666

Bambang Parmanto, PhD
Professor
University of Pittsburgh
Dept. of Health Information Management
School of Health & Rehabilitation Science
6026 Forbes Tower
Pittsburgh, Pennsylvania 15260
Telephone: 412-383-6649

Roxanna Bendixen, PhD, OTR/L
Assistant Professor
University of Pittsburgh
Dept. of Occupational Therapy
School of Health & Rehabilitation Science
5025 Forbes Tower
Pittsburgh, Pennsylvania 15260
Telephone: 412-383-6603

SOURCE OF SUPPORT: National Institute on Disability and Rehabilitation Research

Why is this research being done?

Researchers involved with this project are interested in improving technology that they have developed to help caregivers support their dependent in managing their medical problems. By better understanding chronic conditions and disability, and opinions of potential users of the system, researchers can then work to improve the software. The purpose of this study is to find out what features and functions of the technology would best support caregivers in assisting the person they care for prepare for adult life and take better care of themselves.

Who is being asked to take part in this research study? You are being invited to take part in this research study because you are the primary caregiver of an individual with a disability or chronic health conditions. We would like you to take part in a discussion with other caregivers on how to best support and prepare individuals with disabilities and chronic conditions to take better care of themselves using technology.

What procedures will be performed for research purposes?

If you decide to take part in this research study, you will be asked to take part in a group discussion with other caregivers, fill out a questionnaire and/or participate in an interview. You may be asked to review a mobile health support system and provide feedback for improvements.

The session will take about <u>60 minutes</u>. The sessions will be audio recorded and later transcribed. This audio recording will allow us to obtain more detailed information about your responses than the handwritten notes that will be taken by investigators, and it will allow us to double check our data for accuracy. Basic information about you such as age and medical problems of your dependents will be collected. The system will be revised based on your feedback and in the future you may be asked to participate in another round of testing. A member of the research team may call you to clarify any information that is missing or unclear.

University Of Pittsburgh Institutional Review Board **Approval Date: 2/26/2016** **Expiration Date: 2/25/2017** **IRB #: PRO14120443**

We want to protect your privacy, so we will not send your name, address, telephone number or other personal identifiable information to anyone. Your personal data will remain protected within the Department of Physical Medicine and Rehabilitation, University of Pittsburgh and will only be accessed by trained members of the research staff.

Neither you, nor your insurance provider, will be charged for the costs of any of the procedures performed for the purpose of this research study. You will be charged, in the standard manner, for any procedures performed for your routine medical care.

What are the possible risks, side effects, and discomforts of this research study?
The risks of participating in this research are minimal. You may experience fatigue when answering questions in the interviews or during the group discussion. You will be allowed to take breaks during the interview.

The discussion will be kept *strictly confidential*. The other caregivers in the group will be asked to keep private what we talk about in the discussion group, but this cannot be guaranteed. There is also a risk of accidental release of your information (a breach of confidentiality); however, we will make every effort to prevent this from happening.

Who will know about my participation in this research study?
Any information we gather about you from this research study will be kept as confidential (private) as possible. To keep your information private, your name will not be used in any publication of research results unless you sign a separate release form giving your permission. In unusual cases, your research records may need to be released in response to an order from a court of law. If the researchers learn that you or someone with whom you know is involved in serious danger or harm, they will need to inform the appropriate agencies as required by Pennsylvania law. Other people who may know of your participation are other caregivers in the discussion group, and medical staffs that work in the clinic where the person you care for is seen. This would include people such as nurses, nursing assistants and the medical secretaries. It is also possible that authorized representatives of the University of Pittsburgh Research Conduct and Compliance Office may inspect your research records as part of the institution's routine oversight of research.

The information that we gather about you on paper, such as this consent form, or audio files recorded on tape will be kept in a locked cabinet in the Department of Physical Medicine and Rehabilitation at the University of Pittsburgh. Your research files will be assigned a specific identification number or case number. The information linking the case number to your personal information will be stored in a separate, locked location within the Department of Physical Medicine and Rehabilitation. Any electronic information, (information gathered about you and typed into a computer or electronic audio files), that does not contain your personal and identifiable information will be kept by the Department of Health Information Management at the University of Pittsburgh. The electronic information will be stored on restricted access, password protected servers. Trained researchers, who are directly working on this project, will have access to your personal information. Your records will be kept for a minimum of seven years after final reporting or publication of the project.

Your research data may be shared with investigators conducting similar research. However, this information will be shared in a de-identified manner.

What are possible benefits from taking part in this study?
There are no direct benefits for your participation in this research study. You may, however, gain the satisfaction of knowing that the information gained from this research could help to improve the healthcare of individuals with chronic conditions and disabilities, and their caregivers. However, such a benefit cannot be guaranteed.

Will I be paid if I take part in this research study?
You will receive $25 as compensation for your time and effort to participate in this study.
You may be asked to participate in future rounds of the study. You will be separately compensated for your participation in each round of the study in the same terms described above.

Is my participation in this research study voluntary?
Your participation in this research study is completely voluntary. Whether or not you decide to participate in this research study will have no effect on your current or future relationship with the University of Pittsburgh, or your current or future medical care at

University Of Pittsburgh Institutional Review Board **Approval Date: 2/26/2016** **Expiration Date: 2/25/2017** **IRB #: PRO14120443**

a UPMC hospital or affiliated health care provider, or your current or future relationship with a health care insurance provider. If there is any question that makes you feel uncomfortable, you do not have to answer them. You may leave the group discussion at any time for any reason.

May I withdraw, at a future date, my consent for participation in this research study?
Your participation in this research study is completely voluntary. You do not have to take part in this research study and, should you change your mind, you can withdraw from the study at any time. Your current and future care at a University of Pittsburgh Medical Center, and your current and future relationship with the University of Pittsburgh and any other benefits for which you qualify will be the same whether you participate in this study or not.

If I agree to take part in this research study, can I be removed from the study without my consent?
It is possible that you may be removed from the research study by the researchers if, for example, it is determined that you no longer qualify, or if you do not follow the instructions of the researchers.

Disclosures
One or more of the investigators conducting this research has a financial interest in the software system being evaluated in this research study. This means that it is possible that the results of this study could lead to personal profit for the individual investigator(s) and/or the University of Pittsburgh. Any questions you might have about this will be answered fully by the Principal Investigator, Dr. ***Andi Saptono, at 412-383-5101,*** who has no financial conflict of interest with this research, or by the Human Subject Protection Advocate of the University of Pittsburgh (866-212-2668).

VOLUNTARY CONSENT
All of the above has been explained to me and all of my current questions have been answered. I understand that I am encouraged to ask questions about any aspect of this research study during the course of this study, and that such future questions will be answered by the researchers listed on the first page of this form. Any questions I have about my rights as a research participant will be answered by the Human Subject Protection Advocate of the IRB Office, University of Pittsburgh (1-866-212-2668).

By signing this form, I agree to participate in this research study. A copy of this consent form will be given to me.

______________________________ _________________
Printed Name of Participant Date

____
Participant's Signature

****

<u>CERTIFICATION OF INFORMED CONSENT</u>:
I certify that I have explained the nature and purpose of this research study to the above-named individual, and have discussed the potential benefits and possible risks of study participation. Any questions the individual has about this study have been answered, and we will be available to address future questions as they arise.

______________________________ ________________________
Printed Name of Person Obtaining Consent Role in Research Study

_________________________________ _______________________
Signature of Person Obtaining Consent Date
