# Supplementary material for: A User-Centered Approach: Understanding Client and Caregiver Needs and Preferences in the Development of mHealth Apps for Self-Management
Source: JMIR Mhealth Uhealth. 2017 Sep 26;5(9):e141. doi: 10.2196/mhealth.7136 (PMC5635231; doi:10.2196/mhealth.7136)
Supplement: Multimedia Appendix 3 [file mhealth_v5i9e141_app3.pdf]

<u>Example of Focus Group Discussion Guide (Youth)</u>

MHealth Self-Management and Support System for Chronic and Complex Health Conditions (Phase 1-Development and Refinement)

---

1) Welcome, consent process, and introduction

a. <u>Welcome</u>

Good morning/afternoon/evening and welcome to our session.
Thanks for taking the time to join us and to talk about promoting independence and self-management using mobile health technology – or mHealth. My name is ______________ and assisting me is ____________. The goal of this study is to further develop and test a mobile health system that we developed to support self-management of individuals with chronic and complex health conditions. Mobile health is basically the use of mobile devices, like smartphones, to help persons become healthier or stay healthy. We are meeting with persons, like yourselves, to better understand what you would find helpful. We would like to talk today to explore your needs and preferences for mHealth. We also would like to discuss and identify mobile health applications that would be important to support your self-care routines. We will show you a mHealth system we have developed. We are seeking your thoughts and ideas to help us make the mHealth technology even better, especially for teens, young adults and their families.

b. <u>Consent Process</u>

As a group, we are going to go over the informed consent form before we start our focus group to be sure that you understand why we are having this focus group and to be sure that you voluntarily want to participate.

c. <u>About the focus group</u>

- Ask the group if anyone has participated in a focus group before. Explain that focus groups are being used more and more to gain information to further inform future trials, study planning, etc.
- You are the experts: we learn from you (positive and negative). Individuals with chronic and complex health conditions are being invited to participate because you have important knowledge about particular experiences, needs, or perspectives that we hope to learn more about as a result of our discussion today in this focus group.
- We are not trying to get everyone to agree or achieve consensus, rather, we're gathering information. It is okay if you have different opinions and ideas than the other persons in the group. It is our goal to develop a patient-centered mHealth device that will assist you in being more independent in taking care of your health. We want the technology to meet your individual needs and preferences.

2) Focus group logistics and ground rules

a) <u>Logistics</u>

- Focus group will last about one hour (60 minutes)
- Feel free to move around
- Where is the bathroom? Exit?
- Help yourself to refreshments

b) <u>Ground Rules</u>

- Everyone should participate and only one person talks at a time
- It is important for us to hear everyone’s ideas and opinions
- There are no right or wrong answers to questions – just ideas, experiences and opinions, which are all valuable
- The session will be audio recorded to help us gather more detailed information about your responses than the handwritten notes that will be taken by investigators, and it will allow us to double check our data for accuracy (if session is audio recorded, which is depending on consent from individual participants).
- Stay with the group, please don’t have side conversations, and speak clearly to increase recording quality
- Turn off or silence cell phones.
- Enjoy the discussions

Ask the group if there are any questions before we get started, and address those questions.

Turn on Tape Recorder

Focus Group Questions

Discussion begins, make sure to give people time to think before answering the questions and don’t move too quickly.

<u>Opening Question (round robin)</u>

1. Tell me about your experience with using a smartphone. Are there any apps that you currently have on your smartphone that you like and use often?

Probes may include the following apps:

Games
Communication
Education
Tools
Transportation and Travel
Weather
Time Management
Books, Magazines, and News
Entertainment (Music, Movie….)
Social Network
Shopping or To-Do list
Health and Fitness
Photo and Video

2. Are there any apps that you currently use but DO NOT like? What are the apps? Please tell me what you don’t like about it (them).

3. Can you tell me your experience with mobile health (mHealth) technology? Please give me an example of how you have used applications to support or manage your health.
   In this discussion, when we talk about mHealth technology, we use it to refer to the use of mobile technology such as a smartphone and tablet for supporting the delivery of healthcare services or for self-care of one’s health conditions.

<u>Introduce the current version of our mHealth system here.</u>

We would like to show you the mobile health system that we have developed to support self-care for individuals with chronic and complex conditions. The mHealth system currently has 5 apps that can be personalized and tailored to an individual’s needs. This means that each person would only use the apps that he or she needs. These are the five apps we have created so far: medication, skincare, bowel management, self-catheterization, and mood monitoring. The apps also allow the person to personalize times for reminders to do the things they need to in order to stay healthy, like taking medications on time. More apps for different kinds of needs can be created and added or replace these apps, so that the system works for each person’s unique health needs.

4. What are some applications you feel you could use to be more independent in taking care of your health? Why?

5. Talk to me about how you could use a mobile health device, such as the one we have developed, and the applications that are on the device. What about the device would make it difficult or easy to use?

6. Can you tell me what resources you need or could use to better assist you with self-managing your health? For example, educational materials to help you or your caregiver better understand your diagnosis, or reminders to make a doctor’s appointment or take your medications. Are there other resources you can think of that would help you better take care of yourself?

7. Do you think it is helpful to be able to access _________, ___________, from your mHealth device? Why?

8. What would motivate you to use this type of mHealth technology? For example, what would make it interesting so that you would use this technology every day?

9. What would stop you from using this type of mHealth technology?

10. What would be your concerns in using an mHealth technology like this to help you manage your daily routine?

Ending Questions on mHealth Design & Development

11. If you could change anything about the current mHealth system that we showed you today, what would you change and why? *Probes may include the following: additional features or apps, change in setting or appearance, etc.*

12. Is there anything further you would like to discuss that we did not ask you regarding the use of a mHealth system for self-management of your health and wellness?

Questions on Privacy and Security

****

That concludes our focus group. Thank you so much for coming and sharing your thoughts and opinions with us. We would like for you to complete our focus group survey.

****
